# Metabolic Adaptation in Epilepsy: From Acute Response to Chronic Impairment

**DOI:** 10.3390/ijms25179640

**Published:** 2024-09-06

**Authors:** Agustin Liotta, Stefan Loroch, Iwona Wallach, Kristoffer Klewe, Katrin Marcus, Nikolaus Berndt

**Affiliations:** 1 Department of Anesthesiology and Intensive Care, Charité—Universitätsmedizin Berlin, corporate member of Freie Universität Berlin and Humboldt-Universität zu Berlin, 10117 Berlin, Germany; agustin.liotta@charite.de; 2 Institute of Neurophysiology, Charité—Universitätsmedizin Berlin, corporate member of Freie Universität Berlin and Humboldt-Universität zu Berlin, 10117 Berlin, Germany; 3 Institute of Computer-Assisted Cardiovascular Medicine, Deutsches Herzzentrum der Charité (DHZC), 13353 Berlin, Germany; iwona.wallach@dhzc-charite.de; 4 Department of Experimental Neurology, Charité—Universitätsmedizin Berlin, corporate member of Freie Universität Berlin and Humboldt-Universität zu Berlin, 10117 Berlin, Germany; 5 Medizinisches Proteom-Center, Center for Protein Diagnostics (PRODI), Medical Faculty, Ruhr-University Bochum, 44801 Bochum, Germany; stefan@loroch.email (S.L.); katrin.marcus@rub.de (K.M.); 6 QC-MS/Fa. Dr. Loroch, BioMedizinZentrum, Otto-Hahn-Straße 15, 44227 Dortmund, Germany; kristoffer.klewe@qc-ms.com; 7 Charité—Universitätsmedizin Berlin, corporate member of Freie Universität Berlin and Humboldt-Universität zu Berlin, 10117 Berlin, Germany; 8 German Institute of Human Nutrition Potsdam-Rehbruecke (DIfE), Department of Molecular Toxicology, 14558 Nuthetal, Germany

**Keywords:** epilepsy, metabolic adaptation, status epilepticus, ATP production, shotgun proteomics

## Abstract

Epilepsy is characterized by hypersynchronous neuronal discharges, which are associated with an increased cerebral metabolic rate of oxygen and ATP demand. Uncontrolled seizure activity (status epilepticus) results in mitochondrial exhaustion and ATP depletion, which potentially generate energy mismatch and neuronal loss. Many cells can adapt to increased energy demand by increasing metabolic capacities. However, acute metabolic adaptation during epileptic activity and its relationship to chronic epilepsy remains poorly understood. We elicited seizure-like events (SLEs) in an in vitro model of status epilepticus for eight hours. Electrophysiological recording and tissue oxygen partial pressure recordings were performed. After eight hours of ongoing SLEs, we used proteomics-based kinetic modeling to evaluate changes in metabolic capacities. We compared our findings regarding acute metabolic adaptation to published proteomic and transcriptomic data from chronic epilepsy patients. Epileptic tissue acutely responded to uninterrupted SLEs by upregulating ATP production capacity. This was achieved by a coordinated increase in the abundance of proteins from the respiratory chain and oxidative phosphorylation system. In contrast, chronic epileptic tissue shows a 25–40% decrease in ATP production capacity. In summary, our study reveals that epilepsy leads to dynamic metabolic changes. Acute epileptic activity boosts ATP production, while chronic epilepsy reduces it significantly.

## 1. Introduction

Epileptic seizures, i.e., paroxysmal, hypersynchronous, and excessive cell firing, are associated with a massive increase in oxygen consumption due to massive perturbance in ion homeostasis and subsequent activation of the Na^+^/K^+^-ATPase [1,2]. Epileptiform activity was shown to go along with drops in partial pressure of tissue oxygen (p_ti_O_2_) and altered NADH/NAD^+^ (nicotinamide adenine dinucleotide) and FADH_2_/FAD (flavin adenine dinucleotide) ratios [3]. In line with the increase in metabolic activity, positron emission tomography studies in humans often show hypermetabolism during epileptic activity [4,5]. Recently, we could quantify the increase in oxygen consumption during seizure-like activity to be fivefold [2]. Despite the increase in metabolic activity during epileptiform activity, interictal phases are characterized by hypometabolism, as manifested by low glucose uptake in FDG-PET [6], and there are multiple explanations for this observed hypometabolism, which are not mutually exclusive but rather coexist, reflecting the (mal-)adaptive changes in energy metabolism. From the supply side, altered expression of transporters for glucose and substrate monocarboxylates (i.e., lactate) have been identified along with an uncoupling of the astrocytic network tunneling for metabolites [7,8,9,10], but very little is known about the cellular metabolic adaptations. 

In general, two basic regulatory mechanisms have been established to regulate metabolic activity to match energetic demand and evade energetic depletion. First, kinetic regulation, describing enzymatic activity changes due to changes in the abundance of substrates, co-factors, or regulators, allows a rapid adaptation of metabolic activity to a varying energetic demand within seconds. This short-term regulation of metabolism includes changes in metabolic intermediates and by-products (such as glucose, glucose 6-phosphate, lactate, oxygen, ATP, ADP, AMP, NADH, NAD, or CO_2_), as well as changes in ion homeostasis (e.g., calcium) or mitochondrial membrane potential [11]. All these changes lead to an activation of the main regulatory enzymes in the glycolytic pathway and citric acid cycle as well as the respiratory chain and oxidative phosphorylation (oxphos) system to increase ATP production to match an increased ATP demand. The second mechanism is to vary the amount of an enzyme, which thereby changes the enzyme’s maximal catalytic capacity. Changes in enzyme amounts can be achieved by changes in the rate of gene transcription, mRNA translation, and protein degradation. For metabolic enzymes, changes in enzyme abundances can only be achieved on the order of hours. While short-term regulation uses available capacities more exhaustively, only long-term regulation can increase the basis for ATP production and thereby enable an adaptation to better deal with similar episodes in the future. 

While the first mechanism explains the increase in metabolic activity during epileptiform activity, it is unknown how metabolic capacities are adapted to the (recurring) metabolic challenges of epilepsy. To elucidate the metabolic adaptations occurring in response to epileptic seizures, we used a rat brain slices model to investigate the acute adaptation of neuronal metabolism in response to ongoing epileptic activity for eight hours. We used a rat model because it allows for controlled experimental conditions that are impossible to achieve in human studies. The use of rat brain slices has been used before for mimicking epileptiform activities under controlled conditions [8].

To explore the effect of chronic epilepsy on metabolic capacities, we used proteomic data from a pilocarpine rat model of mesial temporal lobe epilepsy (MTLE). Furthermore, to check for translation, we used data from patients with temporal lobe epilepsy (TLE) in comparison to non-epileptic controls. 

## 2. Results

### 2.1. Acute Adaptation of Neuronal Slices to Prolonged Seizure-like Activity

**Figure 1:**
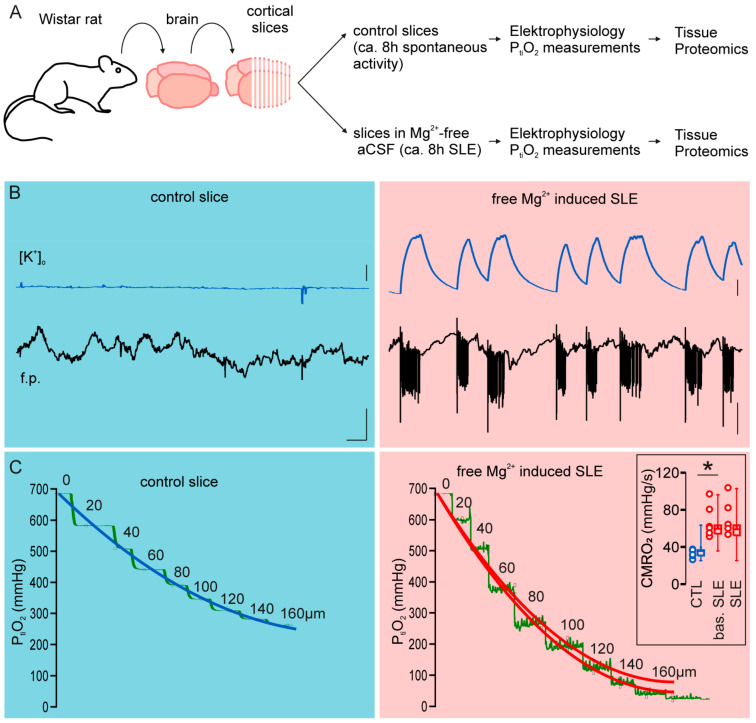
Epilepsy-related changes in cerebral metabolic rate of oxygen (CMRO_2_). (**A**) Graphical representation of the experimental protocol. Cortical slices from one Wistar rat were randomly separated into two groups (control slices maintained for at least 8 h in normal aCSF and slices maintained for the same amount of time in Mg^2+^-free aCSF). (**B**) Slices maintained in normal aCSF displayed spontaneous activity (left, blue background, field potential (f.p.) trace on the bottom in black and extracellular potassium ([K^+^]_o_) on top in blue) and slices treated with Mg^2+^-free aCSF displayed seizure-like events (SLEs, right, red background, field potential trace on the bottom in black and extracellular potassium on top in blue). Scales represent 1 mM in the [K^+^]_o_ trace, 1 mV in the field potential trace, and 10 s, respectively. (**C**) Measurement of tissue O_2_ at different depths (in µm) in control and treated slices. The p_ti_O_2_ profiles show increased oxygen demand in slices with SLEs (right, red background) compared to control slices (left, blue background). In slices with SLEs, pO_2_ measurements display seizure-dependent increases in oxygen consumption, thus basal and SLE-associated CMRO_2_ were calculated (upper and lower red fit line, respectively). Inlet in (**C**): statistical comparison between the control slices and slices with SLE (colors correspond to the fit lines: blue = control, red = SLE). CMRO_2_ was significantly increased in epileptic tissue. * *p*-value < 0.05.

To investigate the acute adaptation of neuronal slices to prolonged seizure-like activity, we separated slices into two groups: a control group maintained in standard conditions (i.e., perfused with artificial cerebrospinal fluid (aCSF)) and a group perfused with Mg^2+^-free aCSF for 8–9 h, respectively. Afterward, we performed electrophysiological and p_ti_O_2_ steps recordings to monitor activity patterns and changes in the cerebral metabolic rate of oxygen (CMRO_2_) for the two groups (Figure 1). In line with our previous works, control slices displayed spontaneous network activity, which was associated with a basal CMRO_2_ of 33.22 ± 4.33 mmHg.s^−1^ (mean ± standard deviation). All slices treated with Mg^2+^-free aCSF displayed seizure-like event (SLE) activity characterized by prolonged tonic–clonic discharges and associated [K^+^]_o_ increases. Measured CMRO_2_ between SLEs (basal CMRO_2_ in treated slices) was 67.80 ± 17.54 mmHg.s^−1^ and slightly increased to 71.38 ± 18.52 mmHg.s^−1^ during SLE activity (*p* = 0.005, paired *t*-test). As expected, CMRO_2_ in slices displaying epileptic activity was significantly increased compared to control slices (*p* < 0.001).

### 2.2. Metabolic Effects

**Figure 2:**
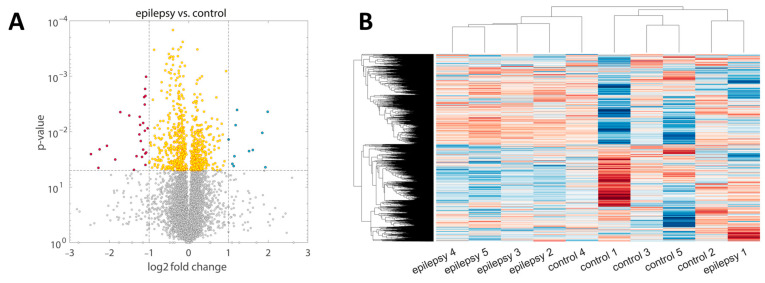
Biostatistics of the global proteome. (**A**) Volcano plot showing the log2 fold changes of all identified proteins (control/epilepsy) concerning the *p*-values. The left side corresponds to proteins that are downregulated, while the right side corresponds to proteins that are upregulated. Red/blue dots show significantly changed proteins (*p*-value < 0.05 and |log2-fold change| > 1/<−1). Only 0.5% of all proteins were significantly differently regulated by that definition. (**B**) Unbiased clustering of the protein abundances of the different samples. The two main clusters each contain four out of five clusters of one group (epilepsy or control), but cluster separation is small. The colors represent normalized values (z-scores) with blue indicating values below the mean and red above the mean.

Biostatistical analysis of proteomic data revealed only minor differences between control samples and samples after 8 h of induced epilepsy (Figure 2). The volcano plot in Figure 2A shows the log2 fold changes of proteins (epilepsy/control) concerning the *p*-values illustrating only small differences in the mean protein intensities. Only 0.5% of the detected proteins showed a significantly different abundance (*p*-value < 0.05, |log2-fold change| > 2). We detected 306 metabolic proteins of which 236 are upregulated in epileptic slices. The mean upregulation was 13%.

Only 37 proteins out of 7071 proteins were significantly changed (*p*-value < 0.05, |log2-fold change| > 2), with no metabolic proteins being significantly differently regulated. To further evaluate differences, we performed a principal component analysis, unbiased protein clustering, and Pearson correlation analysis. Principal component analysis showed that the variance of the samples could not be well explained by their status (control/epilepsy). Only three out of five samples of the epilepsy group separated along the first principal component (Supplementary Figure S1). Clustering of the proteomic profiles of the samples visually led to a slight separation between control and epilepsy, where four out of five samples in each group belong to a cluster (Figure 2B). However, the separation is imperfect and small, again confirming that differences between the groups are minor. Overall, Pearson’s correlation coefficients revealed very high correlations among all samples (R > 0.96). Except for the samples control 1 and epilepsy 1, the correlation among the protein intensity profiles was higher than R > 0.98, showing that differences between samples are extremely small and proteomics sample preparation was of high quality (Supplementary Figure S2).

### 2.3. Metabolic Characterization of Epileptic Brain Tissue 

Overall, biostatistical analysis of proteomic data showed only marginal differences between control and epilepsy tissue, but metabolic functionality is the result of the interplay of a complex interacting network, where small coordinate changes on the protein level can result in significant alterations in metabolic capability. Therefore, to investigate possible metabolic implications of the changes in protein abundance levels, we used kinetic modeling for neuronal energy metabolism to assess metabolic competency and ATP production capacities for each sample separately.

#### 2.3.1. Maximal ATP Production Capacities

**Figure 3:**
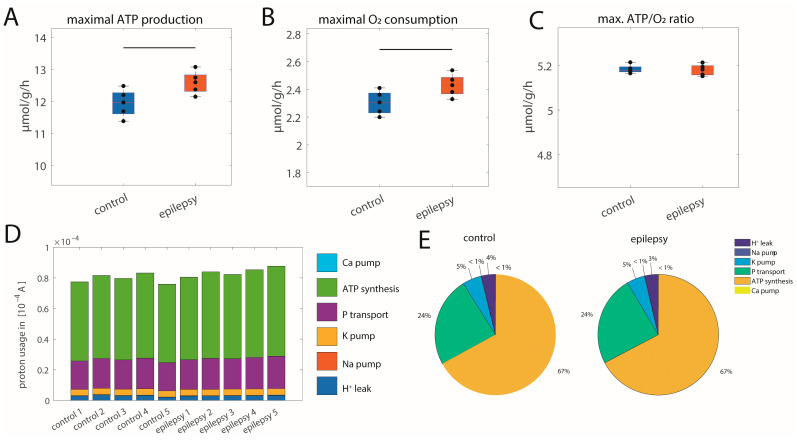
Energy production capacity for control and epilepsy group. (**A**) Maximal ATP production capacity; (**B**) corresponding maximal O_2_ consumption rate; (**C**) corresponding ATP/O_2_ ratio; black dots show values for individual samples. The control group is depicted in blue; the epilepsy group is in orange. The center lines represent the median, the boxes represent the interquartile range, and the whiskers are defined by values within 1.5 times the interquartile range. Black bars indicate significant differences between groups with a *p*-value < 0.05 as assessed with a two-sided *t*-test. (**D**) Proton utilization for the different mitochondrial membrane processes given as currents. The size of the different colored bars indicates the magnitude of the different processes (light blue: calcium pumping; green: FOF1-ATPase; purple: phosphate transport; yellow: potassium pumping; red: sodium pumping; dark blue: proton leakage). (**E**) The mean relative share of the different processes on total proton flux for the two groups.

Maximal ATP production capacities were assessed under physiological conditions. Figure 3 shows that tissue samples exposed to 8 h of induced epilepsy had significantly higher maximal ATP production capacity (A). Mean ATP production capacity was increased by about 10%, which resulted in a corresponding higher maximal utilization of oxygen (B). Importantly, no significant differences in the ATP/O_2_ ratio were found. ATP synthesis relies on the utilization of the proton-motive force generated by the respiratory chain. Figure 3D shows the distribution of the protons pumped by the respiratory chain on the different proton-using processes for each sample at a maximal ATP production capacity. Mitochondrial ion homeostasis including proton leak is not altered in the epilepsy group, but proton utilization for ATP synthesis (*p* = 0.028) and associated phosphate transport (*p* = 0.029) are significantly increased. Figure 3E shows that the relative share of mitochondrial ion homeostasis, FOF1-ATPase, and phosphate transport (which is equivalent to mitochondrial ATP exchange) are not altered, underscoring that increased maximal ATP production capacity does go along with a change in metabolic regulation.

#### 2.3.2. Coordinated Upregulation of Respiratory Chain and Oxidative Phosphorylation Enzymes

Under resting conditions, no significant metabolic differences can be found, and glucose and lactate metabolisms are unchanged (Supplementary Figure S3A,B). In line, the cytosolic redox state, which is one of the main determinants of lactate production (as opposed to utilization of pyruvate in the mitochondria), is consistently higher in the epilepsy group compared to the control group (Supplementary Figure S3C,D).

**Figure 4:**
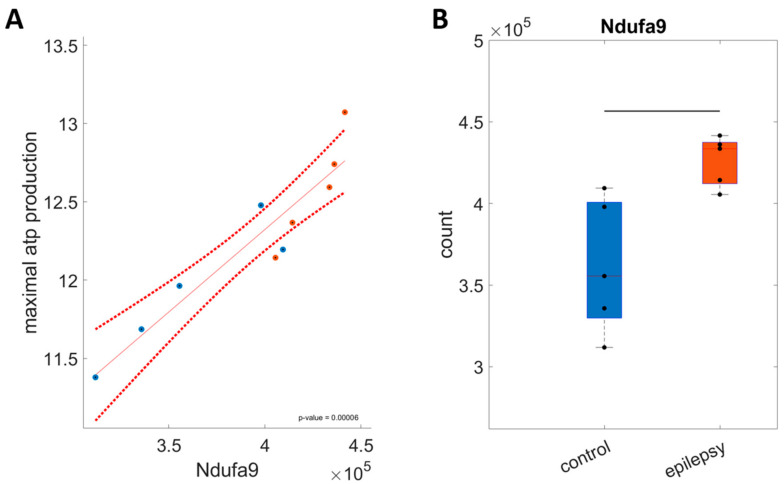
Exemplary relationship between abundance of respiratory chain proteins and maximal ATP production capacity. (**A**) Correlation between Ndufa9, a subunit of complex I, and maximal ATP production capacity; blue dots show abundance levels in individual samples of the control, and orange dots in the epilepsy group (*p*-value = 0.00006 according to the linear regression model with n = 10). Red dotted lines indicate 95% confidence intervals of the linear model. (**B**) Distribution of Ndufa9 abundance in the control and epilepsy group. Corresponding *p*-values = 0.012, based on two-sided *t*-test. The center lines represent the median, the boxes represent the interquartile range, and the whiskers are defined by values within 1.5 times the interquartile range. The black bar indicates a significant difference between groups with a *p*-value < 0.05 as assessed with a two-sided *t*-test.

The unchanged ATP/O_2_ ratio, the higher redox state at high ATP demand, and the unchanged utilization of glucose under resting conditions all indicate that the changes in maximal ATP production capacity are not due to a selective increase in glycolytic activity but rather a coordinated increase in the capacity of the respiratory chain and oxidative phosphorylation. To better understand the underlying enzymatic pattern leading to the increased ATP production capacity, we identified 58 out of 105 proteins belonging to the respiratory chain and the oxphos system that significantly correlate with the ATP production capacity. Figure 4 shows an example of Ndufa9, a subunit of complex I of the respiratory chain. As can be seen in Figure 4A, an increase in Ndufa9 is strongly associated with maximal ATP production (*p* = 0.00006). Figure 4B shows the distribution of Ndufa9 in the individual samples across the two groups. There is a significantly higher abundance in the epilepsy than in the control group. The corresponding data for all 58 proteins can be found in the Supplementary Table S1. Out of the 58 proteins, only one (Ak5) is negatively associated and 57 are positively correlated with the maximal ATP production capacity. Ak5 is the adenylate kinase 5, catalyzing the equilibrium between ATP, ADP, and AMP, important for the indication of ATP availability but not per se for ATP production. Of the significantly associated proteins, 34 are significantly increased in the epilepsy group and only Ak5 is significantly decreased (*p* < 0.05), with another nine proteins tending to be increased in the epilepsy group (*p* < 0.1). This shows that increased ATP production capacity is the effect of a highly coordinated upregulation of enzymes belonging to the respiratory chain and the oxphos system.

#### 2.3.3. Metabolic Changes in Chronic Epileptic Tissues

So far, we have looked at metabolic changes as a response to acute epilepsy-induced energetic challenges. However, chronic epileptic tissue periodically experiences these energetic challenges for years and the central question is if long-term adaptation resembles the observed short-term changes. To answer this question, we used a public dataset from a pilocarpine rat model of MTLE [12]. 

**Figure 5:**
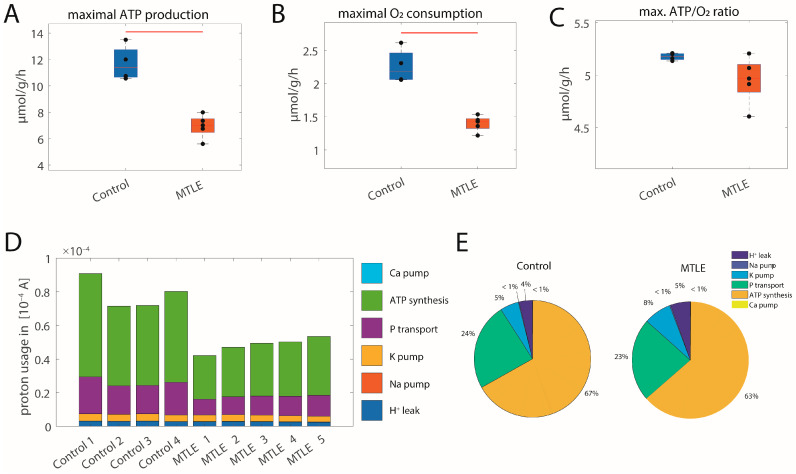
Energy production capacity for control and epilepsy groups in a pilocarpine rat model [12]. (**A**) Maximal ATP production capacity; (**B**) corresponding maximal O_2_ consumption rate; (**C**) corresponding ATP/O_2_ ratio; black dots show values for individual samples. The control group is depicted in blue; the epilepsy group is depicted in orange. The center lines represent the median, the boxes represent the interquartile range, and the whiskers are defined by values within 1.5 times the interquartile range. Red bars indicate significant differences between groups with a *p*-value < 0.001 as assessed with a two-sided *t*-test. (**D**) Proton utilization for the different mitochondrial membrane processes given as currents. The size of the different colored bars indicates the magnitude of the different processes (light blue: calcium pumping; green: FOF1-ATPase; purple: phosphate transport; yellow: potassium pumping; red: sodium pumping; dark blue: proton leakage). (**E**) The mean relative share of the different processes on total proton flux for the two groups.

Figure 5 shows the metabolic alterations in a pilocarpine rat model of MTLE. As depicted in Figure 5A, the maximal ATP production capacities in the MTLE group are significantly decreased by around 40% (*p*-value < 0.01), as is the maximal oxygen consumption rate (Figure 5B, *p*-value <0.01). The corresponding ATP/O_2_ ratio is also decreased in the MTLE group (Figure 5C), but this is not significant (*p* < 0.1). Figure 5D shows that the relative share of protons used for mitochondrial ion homeostasis is increased in the MTLE group (increasing from ~9% to 13%), but proton utilization for ATP synthesis and associated phosphate transport are severely diminished, indicating mitochondrial dysfunction (Figure 5D,E). In this model, 8 out of 26 proteins of the respiratory chain and the oxphos system are significantly associated with the ATP production capacity (see Supplementary Table S2 for all significantly associated proteins).

To evaluate the translation from rat to human, we used two additional public datasets. The first set is based on proteomic data from two patients with TLE in comparison to two non-epileptic controls recently published [13]. The second dataset is based on transcriptomic data from 14 epileptic tissue samples compared to two controls (https://www.ncbi.nlm.nih.gov/geo/ (accessed on 19 April 2024), accession number GSE134697 [14]). 

**Figure 6:**
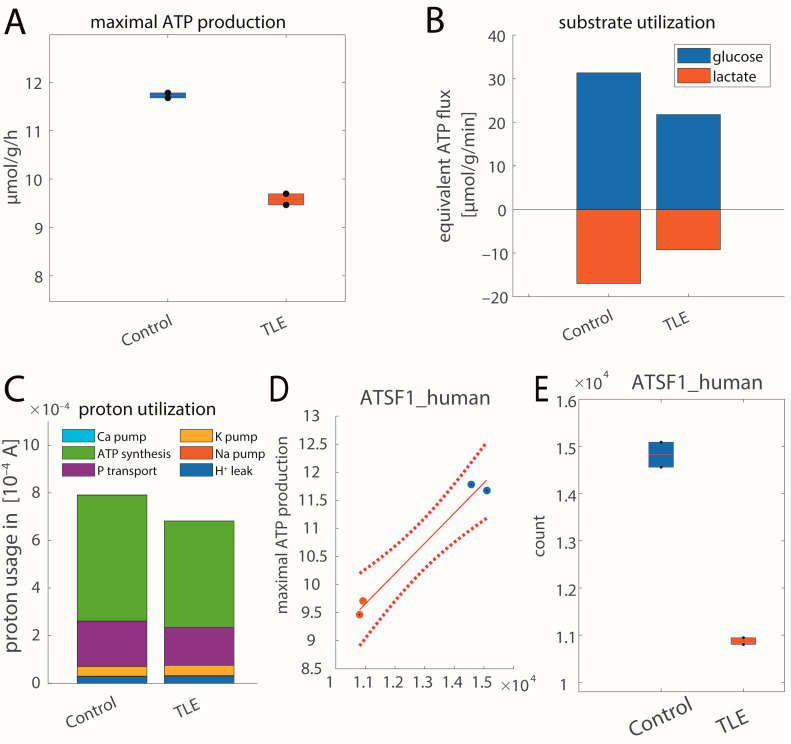
Metabolic alterations in patients with temporal lobe epilepsy (TLE) [13]. (**A**) Maximal ATP production capacity. The control group is depicted in blue, epilepsy group in orange. (**B**) Mean glucose utilization and lactate production rates are decreased in the epilepsy group vs. the control group. Positive values correspond to uptake rates, while negative values depict release rates. The values for the individual samples are given in Supplementary Figure S4A. (**C**) Mean mitochondrial proton utilization for control and epilepsy groups. The size of the different colored bars indicates the magnitude of the different processes (light blue: calcium pumping; green: FOF1-ATPase; purple: phosphate transport; yellow: potassium pumping; red: sodium pumping; dark blue: proton leakage). The values for the individual samples are given in Supplementary Figure S4B. (**D**,**E**) Example of a functional protein significantly associated with ATP production capacity. AT5F1, a subunit of ATP synthase, is significantly associated with ATP production capacity over all samples (*p*-value < 0.01 via linear regression) and is reduced in TLE. The values for the individual samples are given in Table S3.

Figure 6 shows metabolic alterations in patients with TLE. As can be seen in (A), the ATP production capacity in TLE patients is decreased by about 25%, similar to the MTLE rat model. This goes along with a reduced uptake of glucose (B) and a reduced mitochondrial ATP production (C). Underlying these metabolic changes are significant differences in protein abundances between control and TLE tissue. Figure 6D shows significantly associated proteins for ATP production. Overall, 12 out of 95 proteins of the respiratory chain and the oxphos system are significantly associated with the ATP production capacity (see Supplementary Table S3 for all significantly associated proteins).

**Figure 7:**
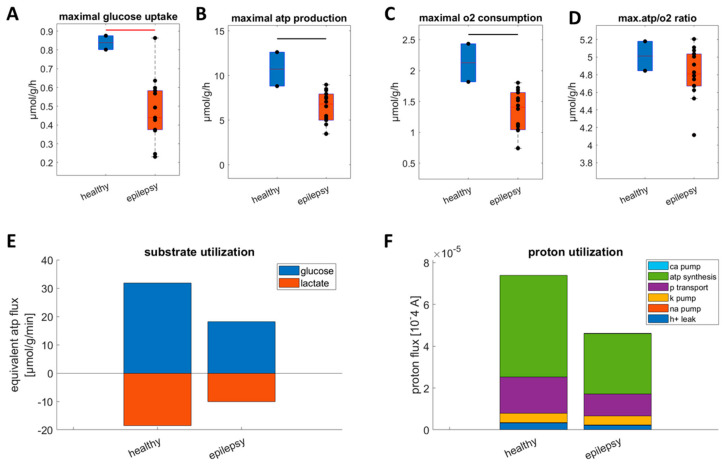
Metabolic alterations in human epileptic tissue [14]. (**A**) Maximal glucose uptake; (**B**) maximal ATP production capacity; (**C**) maximal oxygen consumption rate; and (**D**) ATP/O_2_ ratio at maximal ATP production rate. The healthy group is depicted in blue, and the epilepsy group is in orange. Black and red crossbars indicate significant differences between groups with a *p*-value of <0.05 and <0.01, respectively, as assessed with a two-sided *t*-test. (**E**) The mean glucose utilization and lactate production in control and epilepsy groups at maximal ATP production. Blue bars represent the mean glucose uptake rate in ATP equivalents in each group, while red bars represent lactate efflux in ATP equivalents in each group. The values for the individual samples are given in Supplementary Figure S5A. (**F**) The mean mitochondrial proton utilization for healthy and epilepsy groups at maximal ATP production rate. The size of the different colored bars indicates the magnitude of the different processes (light blue: calcium pumping; green: FOF1-ATPase; purple: phosphate transport; yellow: potassium pumping; red: sodium pumping; dark blue: proton leakage). The values for the individual samples are given in Supplementary Figure S5B.

While the data show a clear downregulation of ATP production capacity, the data are hampered by the very low sample size. Therefore, we analyzed a second published dataset based on transcriptomic data comprising 14 samples of epileptic tissue and two healthy controls. In line with the first dataset, Figure 7 shows a clear downregulation of all relevant metabolic parameters. Glycolytic capacity (A), ATP production capacity (B), and maximal oxygen consumption rate (C) are reduced by around 40%. Interestingly, the energy efficiency measured via the ATP/O_2_ ratio is not significantly changed (D) but the mean utilization of glucose at maximal ATP demand (E) and mean utilization of protons pumped by the respiratory chain (F) show a clear reduction in epileptic tissue. This very much resembles the MTLE rat model. 

A total of 116 out of 135 of the genes of the respiratory chain and the oxphos system are significantly correlated with the ATP production capacity. Out of these 116 genes, 104 are significantly downregulated in the epileptic tissue and not a single one is upregulated. The data for all analyzed mRNAs are given in the Supplementary Table S4. 

## 3. Discussion

In this study, we investigated changes in metabolic capacities in response to 8 h of epileptiform activity in brain slices of Wistar rats in contrast to a pilocarpine rat model of MTLE. Acute SLEs related to Mg^2+^-free medium are pharmacoresistant and generate in the mesial temporal lobe as well [15]. We further compared the rat model to TLE in human tissue. Using proteomics-based molecular resolved metabolic models of neuronal energy metabolism, we found that acute metabolic adaptation leads to an increase in ATP production capacity, while chronic epilepsy results in a downregulation of ATP production capacity. 

Usually, half-life and turnover times for metabolic enzymes are in the range of hours [16]. Therefore, the 8 h interval was chosen because metabolic adaptations, particularly at the protein level, require sufficient time for the stimulus to be effective. Shorter periods would probably be insufficient for measuring changes on the proteomic level, while much longer incubation periods are experimentally hard to control and might introduce more changes in the tissue related to artificial maintenance. Noticeably, metabolic changes on the functional level are in effect immediately as can be appreciated from the instantaneous rise in oxygen consumption rate.

While the observed average changes are small (13%), 77% of the metabolic proteins are upregulated. Of the 63 proteins of the respiratory chain and oxphos system, 36 are significantly upregulated and only 1 is significantly downregulated in epileptic slices. While it is often assumed that key regulatory enzymes carry the burden of metabolic capacity adaptation, our results show that it is not individual enzymes but rather a collaborative effort that drives metabolic adaptation in this case. If the regulations were not occurring in a coordinated manner, we would expect to see a roughly equal number of up- and downregulated enzymes within the oxphos system, which is not observed.

The upregulation of metabolic capacity in response to metabolic and energetic challenges is a uniform feature of many cells and organs. For example, hepatocytes, cardiomyocytes, and skeletal muscle cells all change their metabolic capacities depending on dietary conditions and energy demand (e.g., [17,18,19]). Often, the triggering mechanism for long-term metabolic adaptation are important regulators for short-term adaptation. For example, oxygen depletion activates HIF, a master transcription factor regulating cellular energy metabolism [20]. At the same time, oxygen-dependent energy depletion activates the AMP-dependent kinase immediately regulating energy metabolism via enzyme phosphorylation [21]. In general, the consistent upregulation of a large fraction of metabolic proteins makes it very plausible that physiological adaptation mechanisms are operating.

This acute response, however, is contradicted by the reduction in the ATP production capacity by ~25–40% in an MTLE rat model and patients with epilepsy. In contrast to the acute model, they show a clear downregulation of proteins of the ATP production machinery and a significant mitochondrial dysfunction. While the share of proteins in the respiratory chain and oxphos system pathways significantly associated with ATP production capacity is only 10% compared to 65% in the acute case, this is most likely due to the low sample size, where differences have to be huge to be significant. Indeed, the transcriptomic dataset comprising 14 epileptic tissue samples finds a significant association with ATP production capacity in 84% of the respiratory chain and citric acid cycle proteins. 

It is noteworthy that decreased metabolic capacity is a common feature of diseases in various organs [22,23,24,25,26]. Different mechanisms could be responsible for the chronic loss of ATP production capacity in contrast to its acute stimulation. There is evidence of free radical-dependent disturbances of oxidative metabolism in mesial TLE tissue, in patients with mitochondrial encephalopathies, and in animal models of epilepsy [6,7,27,28,29,30,31,32]. As metabolic capacities strongly depend on nutrient availability, it is worthwhile to put the metabolic alterations during chronic epilepsy in the context of tissue architecture. There is growing evidence that disturbances of the neurovascular unit might contribute to the progression of epilepsy and the observed hypometabolism of the epileptic focus [33,34]. Recurrent epileptic seizures result in disruption of the blood–brain barrier and the subsequent extravasation of plasma contents initiates epileptogenic alterations, including astrocytic transformation, neuroinflammation, excitatory synaptogenesis, and pathological plasticity [34,35]. Also, recurrent seizures may result in neurovascular decoupling due to injury of capillary pericytes [36], and this injury might contribute to the postictal hyporesponsiveness of the vasculature [37]. Pericyte detachment might initiate vascular remodeling and angiogenesis [38,39]. The vascular damage and remodeling would alter the supply–demand relationship during high-energy demanding activity, and it is widely accepted that this potential mismatch could contribute to epileptogenesis [40,41]. This is also in line with the finding that interictal hypoperfusion and hypometabolism have been observed in a wide range of epileptic syndromes including TLE, generalized childhood absence epilepsy, and status epilepticus [42,43,44]. This might imply that hypometabolism during the interictal period reflects the decreased demand of damaged tissue with limited respiratory capacity.

The reduction in metabolic capacity might itself aggravate epileptic activity and thereby contribute to a vicious cycle of epileptiform activity, energy depletion, vascular damage, and reduced nutrient supply. During seizures, extracellular potassium dramatically increases from resting levels [2,45]. As increased extracellular potassium levels are highly excitatory via depolarization of the plasma membrane, the metabolic capability is a prerequisite to re-establish ion homeostasis after epileptiform activity by the Na^+^/K^+^-ATPase, and the decrease of its activity prolongs potassium transients [45]. Therefore, decreased metabolic capacities might underlie the observation that “seizures-beget-seizures” [46]. 

Limitations of this study: this study has a few limitations. First, the sample size for the control group in both human datasets is very small, and data for the acute response to epileptic activity in human tissue are missing. Second, for the human data, we used transcriptomic and proteomic data. These shortcomings have one common ancestry, namely the availability of human tissue, especially in a healthy condition. While this limitation severely hampers statistical analysis and potentially compromises the reliability of the results, it is noteworthy that the human data do not show minor alterations, but the changes in ATP production are in the order of more than 20%, which is a huge effect size that is in concordance with the data from the rat model. Also, the transcriptomic data are in very good agreement with the proteomic data indicating a general phenotype. Furthermore, the consistent downregulation of the vast majority of proteins belonging to the ATP production machinery supports the claim of a coordinated and systematic adaptation rather than random coincidence due to the low sample size. Another important consideration is the mode of adaptation. While metabolic adaptation at the cellular level is influenced by various factors such as nutrient availability, ion homeostasis, synaptic activation, and waste products [47,48,49], the microenvironment in vivo may differ from that in vitro. For instance, hypoxic regions are likely to vary between in vivo and in vitro settings due to the regulation of blood flow and changes in supply–demand relationships. Moreover, metabolic adaptations that occur at the structural level, such as changes in vascular density or perfusion rate, cannot be accurately replicated in an in vitro model.

Future perspectives: to determine whether this observed effect is a general characteristic of human epilepsy, future studies should examine the acute response of human brain tissue to epileptiform activity. An important issue in this domain is to differentiate hypometabolism from mere neuronal death and gliosis [50,51]. Additionally, investigating the long-term consequences of these metabolic adaptations, including potential downstream effects on neuronal function and survival, could provide deeper insights into the progression of epilepsy. Expanding this study to include a broader range of epilepsy models, as well as incorporating advanced imaging techniques to monitor metabolic changes in real time, would also be valuable for validating and extending these findings.

Conclusions: We found that during acute seizure-like events, neuronal tissue upregulates its ATP production capacity, reflecting an adaptive metabolic response to increased energy demands. In contrast, chronic epileptic tissue shows a significant decrease in ATP production capacity, suggesting a long-term impairment in metabolic function. These findings suggest distinct metabolic adaptations in epilepsy, with acute responses aimed at energy compensation, while chronic conditions lead to severe metabolic decline.

## 4. Materials and Methods

### 4.1. Slice Preparation, Maintenance, and Induction of Seizure Activity

This study complies with the ARRIVE 2.0 guidelines, the Helsinki Declaration, and the Charité animal welfare guidelines. Animal use was approved by the State Office of Health and Social Affairs of Berlin (Permission T-CH 0014/23). For in vitro experiments, horizontal hippocampal slices containing the entorhinal cortex were prepared from one male Wistar rat (weight: 200 g, age: 8 ± 1 weeks) as previously described [52]. Slices were transferred and maintained in a custom built interface chamber. aCSF gassed with carbogen (95% O_2_ and 5% CO_2_, from Linde, München, Germany) contained (in mM) the following (all from Merck, Darmstadt, Germany): 129 NaCl, 21 NaHCO_3_, 10 glucose, 3 KCl, 1.25 NaH_2_PO_4_, 1.6 CaCl_2_, and 1.2 MgCl_2_. The osmolarity was 295–305 mosmol/L, and the pH was 7.35–7.45. To avoid possible bias due to time of slicing, macroscopic properties, or others, we first prepared all slices and then randomly distributed the slices from one pool into two incubation chambers. After a recovery time of two hours in similar conditions, slices were separated into two groups: a control group maintained for ca. 8–9 h in normal aCSF and a treated group maintained for ca. 8–9 h in Mg^2+^-free aCSF. We used a timeframe of 8 h to have a chance to see measurable alterations in metabolic protein abundance as metabolic adaptation takes time on the protein level. 

All slices treated with Mg^2+^-free aCSF display SLEs typically characterized by paroxysmal field potential discharges and related extracellular potassium [K^+^]_o_ rises. NMDA-receptor activation and impairment of GABAergic transmission are the main accepted mechanisms of SLEs in this model. Mg^2+^-free associated SLEs are also resistant to clinically used antiepileptic drugs, which is typically observed in the most common type of epilepsy in humans (i.e., TLE) [15]. At the end of the experiments, slices were separately snap-frozen and stored at −80 °C before proteomic analysis. 

### 4.2. Electrophysiology and p_ti_O_2_ Recordings

Field potential and [K^+^]_o_ were measured in the entorhinal cortex using double-barreled ion-sensitive microelectrodes constructed and calibrated as reported by Liotta et al. [52]. The Potassium Ionophore I 60031 (Fluka, Buchs, Switzerland) was used accordingly. Changes in p_ti_O_2_ were measured with Clark’s type probes (Unisense, Aarhus, Denmark). To measure changes in p_ti_O_2_, the O_2_ electrode was moved vertically through the slice in 20 μm steps until reaching the minimum of p_ti_O_2_. The CMRO_2_ was calculated as described below. Recordings were performed after eight hours of each treatment. We reduced variability by recording slices from one animal and one preparation. Moreover, we performed only one measurement after a long period of ongoing activity or control conditions to reduce tissue stress due to more manipulation and electrode-related cell injuries.

### 4.3. Data Acquisition and Data Analysis

Analog signals were digitalized with Power CED1401 and Spike2 software (both from Cambridge Electronic Design, Cambridge, UK). Data analysis and statistics were performed using Spike2, Excel 2021 (Microsoft, Seattle, WA, USA), and Origin (Version 6, Microcal Software, Northampton, MA, USA). Concerning CMRO_2_, the median values and corresponding 25th and 75th percentile in brackets are described in the results. Data are shown in box plots (with median, mean, and 25th and 75th percentile). CMRO_2_ in control slices (without treatment) and slices treated with Mg^2+^-free aCSF were compared using an independent *t*-test. Changes were stipulated to be significant for *p*-values < 0.05.

### 4.4. Calculation of Cerebral Metabolic Rate of O_2_

As previously described, the calculation of CMRO_2_ was performed from p_ti_O_2_ depth profiles [53]. In short, we applied a reaction–diffusion model for O_2_ consisting of diffusive O_2_ transport and consumption within the slice. Slices were divided into layers with equal thickness of 1 μm. The diffusive distribution of O_2_ between the layers is described by Fick’s Law with a diffusion constant of 1.6 × 10^3^ μm^2^/s and O_2_ consumption rate within each layer is given by Michaelis–Menten kinetics with a K_m_ value of 3 mmHg [54]. The CMRO_2_ was assumed to be homogeneous throughout the slice and is treated as an adjustable parameter to match the experimental data. For the boundary conditions, the p_ti_O_2_ concentration at the slice surface was fixed to the supply value, while at the p_ti_O_2_ minimum, the diffusive transport of O_2_ was put to zero.

### 4.5. Proteomics Sample Preparation

Solvents and chemicals for LC-MS-based proteomics were purchased from Merck Darmstadt, Germany, unless otherwise stated. For proteomics analysis, the snap-frozen slices (see Section 4.1) were lysed in 10% sodium dodecyl sulfate (*w*/*v*), pH 7.8, including one tablet cOmplete Mini and one tablet PhosSTOP (both from Roche, Basel, Switzerland) per 10 mL. Samples were homogenized using 30 s cycles of ultrasound (Vial Tweeter, Hielscher Ultrasonics, Teltow, Germany), repeated six times, followed by centrifugation at 18,000× *g* at 4 °C for 15 min (benchtop centrifuge 5417R, Eppendorf, Hamburg, Germany). The intermediate phase was transferred to a new tube. Protein concentrations were determined using a bicinchoninic acid assay (Pierce, Thermo-Fisher Scientific, Bremen, Germany). Cysteines were reduced by adding dithiothreitol to a final concentration of 10 mM and incubation for 30 min at 56 °C followed by alkylation of the free sulfhydryl groups by adding iodoacetamide to a final concentration of 20 mM and incubation for 30 min at RT in the dark. Samples were processed via suspension trapping using S-Trap mini cartridges (ProtiFi, Fairport, New York, NY, USA) according to vendor instructions. In brief, samples were acidified with phosphoric acid to a final concentration of 1.2% and subsequently diluted 1:10 with S-Trap loading buffer, comprising 50 mM triethylammonium bicarbonate (TEAB), pH 7.55, and 90% MeOH. Samples were loaded onto the S-Trap cartridges and washed three times with 400 µL loading buffer at 2000× *g* for 2 min using a benchtop centrifuge. Digestion was performed in 125 µL of 50 mM TEAB and 1 mM CaCl_2_, and trypsin (Serva, Sequencing Grade Modified, Heidelberg, Germany) was added in an enzyme-to-protein ratio of 1:10 (*w*/*w*). After incubation for 14 h at 37 °C, peptides were recovered in three centrifugation steps using (I) 80 µL of 50 mM TEAB, (II) 80 µL of 0.2% formic acid (FA), and (III) 80 µL of 50% acetonitrile (ACN). After drying the eluate in a vacuum centrifuge (concentrator plus, Eppendorf, Hamburg, Germany), peptides were redissolved in 0.1% trifluoroacetic acid for LC–MS.

### 4.6. Liquid Chromatography–Mass Spectrometry (LC–MS)

Samples were analyzed using an Exploris 480 MS online coupled to a Vanquish Neo nanoLC (both from Thermo Scientific including columns, Dreieich, Germany) equipped with a PepMap Neo Trap Cartridge (0.3 × 5 mm, 5 µm particles) and a DNV PepMap Neo main column (0.075 × 50 mm, 2 µm particles). Peptides were loaded onto the trap column in 0.1% trifluoroacetic acid at 60 µL/min using a loading volume of 20 µL. Peptides were separated on the main column at a flow rate of 400 nL/min using a stepped linear gradient from 1 to 16% acetonitrile (ACN) in 80 min and from 16 to 30% ACN in 40 min in the presence of 0.1% formic acid. The MS was operated in data-independent acquisition, using a survey with a resolution of 120,000 followed by 59 MS/MS scans spanning the mass range of 380–980 *m*/*z* using a resolution of 15,000 and an isolation width of 11 *m*/*z* with 1 *m*/*z* overlap between windows. The normalized collision energy was set to 30%, the maximum Automatic Gain Control (AGC) target value was set to 2000%, and the max injection time mode was set to auto. The polysiloxane signal at *m*/*z* 445.12003 was used as an internal calibrant.

### 4.7. Generation of the Spectral Library 

To increase the depth of analysis, a spectral library was generated using tip-based high-pH-RP fractionation. A total of 50 µg of a pooled peptide sample were loaded in 10 µL 10 mM ammonium formate, pH 9, onto a Hypersep 10–200 µL C18 SpinTip (Thermo Scientific, Bremen, Germany). Peptides were eluted in 45 steps of 20 µL with a 1.4% increase in ACN with each step (in the presence of 10 mM ammonium formate, pH 9.5) to generate ten-peptide fractions via concatenation. Then, 20% of each fraction was analyzed using the same LC setting but with the MS operated in data-dependent acquisition mode. Survey scans were acquired at a resolution of 120,000 using a normalized AGC target value of 250% and a max ion injection time of 150 ms, and the 25 most intense precursors with a charge state ≥ +2 were fragmented via HCD using a normalized collision energy of 31%. MS/MS was acquired using a resolution of 30,000, a normalized AGC target value of 150%, and a maximum ion injection time of 50 ms. Precursors were excluded from re-fragmentation for 80 s (dynamic exclusion). 

### 4.8. Data Analysis

All data-independent acquisition runs including spectral library runs were analyzed using the default setting of Spectronaut v16 (Biognosys AG, Zurich, Switzerland) and the UniProt taxonomy Rattus norvegicus database with duplicate sequence entries removed (11-2023, 26,314 target sequences).

### 4.9. Metabolic Modeling

Metabolic pathways: the kinetic model comprises the major cellular metabolic pathways of mitochondrial energy metabolism and glycolysis [11]. The model also contains key electrophysiological processes at the inner mitochondrial membrane including the membrane transport of various ions, the mitochondrial membrane potential, and the generation and utilization of the proton-motive force. The time-dependent variations in model variables (i.e., the concentration of metabolites and ions) are governed by first-order differential equations. Time variations of small ions were modeled with kinetic equations of the Goldman–Hodgkin–Katz type. Numerical values for kinetic parameters of the enzymatic rate laws were taken from reported kinetic studies of the isolated enzymes. Maximal enzyme activities (V_max_ values) were estimated based on functional characteristics and metabolite concentrations of healthy neuronal tissue [11].

Individual model parametrization: individual metabolic models were established by using the protein intensity profiles delivered via quantitative shotgun proteomics to scale the maximal activities of enzymes and transporters, thereby exploiting the fact that the maximal activity of an enzyme is proportional to the abundance of the enzyme protein according to the following relation:

(1) $$v_{max}^{sample}=v_{mx}^{mean\;control}\frac{E^{sample}}{E^{mean\;control}}$$

The maximal activities $v_{mx}^{mean\;control}$ for the control were obtained from [11]. $E^{mean\;control}$ denotes the mean protein abundance in the control group, and $E^{sample}$ denotes the protein abundance of enzyme E in the sample.

Evaluation of energetic capacity: energetic capacity was assessed under conditions of saturating glucose and oxygen concentrations, which correspond to healthy physiological states. Energetic capacities were evaluated by calculating the changes in the metabolic state induced by an increase in the ATP consumption rate above the resting value. The ATP consumption rate was modeled using a generic hyperbolic rate law

(2) $$v_{ATP}=k_{load}\cdot \frac{ATP}{ATP+K_{m}}$$

The parameter $k_{load}$ was stepwise increased until the ATP production rate reached its maximum value.

### 4.10. Statistical Analysis

Statistical analysis of proteomic data was performed using a two-sample *t*-test. Significantly regulated proteins between epileptic groups and controls were indicated in Tables S1–S4. Hierarchical clustering analysis was conducted using MATLAB’s clustergram function (R2023b, The MathWorks, Inc., Natick, MA, USA) to explore patterns in the proteomic dataset. The clustergram function was applied to the dataset to generate a heatmap with hierarchical clustering of both rows (proteins) and columns (samples). 

For group comparison, data were checked for normality with a one-sample Kolmogorov–Smirnov test. Significant differences between groups were assessed with a two-sided *t*-test for normal distributed group values; otherwise, the Wilcoxon signed-ranked test was used. Statistical and cluster analysis were performed with MATLAB’s bioinformatics toolbox. 

To assess the relationship between the ATP production capacity and expression/abundance levels of metabolic enzymes, a linear regression model was employed using MATLAB’s fitlm function. This function fits a linear model to the data by estimating the coefficients through least squares minimization. The statistical significance of the predictors was determined using *p*-values, with a threshold set at 0.05.

## Data Availability

The mass spectrometry proteomics data have been deposited to the ProteomeXchange Consortium via the PRIDE [55] partner repository with the dataset identifier PXD055090.

## Supplementary Materials

The following supporting information can be downloaded at: https://www.mdpi.com/article/10.3390/ijms25179640/s1.
