# Supplementary material for: Metabolic Adaptation in Epilepsy: From Acute Response to Chronic Impairment
Source: Int J Mol Sci. 2024 Sep 6;25(17):9640. doi: 10.3390/ijms25179640 (PMC11395010; doi:10.3390/ijms25179640)
Supplement: Supplementary file 1 [file ijms-25-09640-s001.zip › Supplementary Figures.pdf]

Supplementary Figures

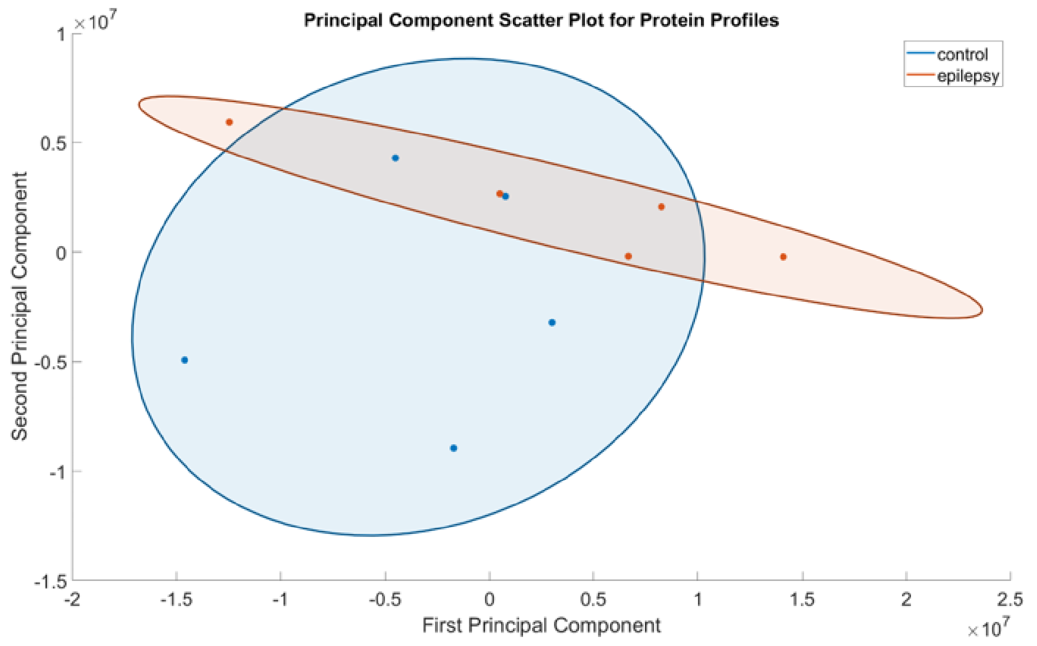

Figure S1. Principal component analysis shows no clear separation between the control samples and epilepsy samples along the first two principal components.

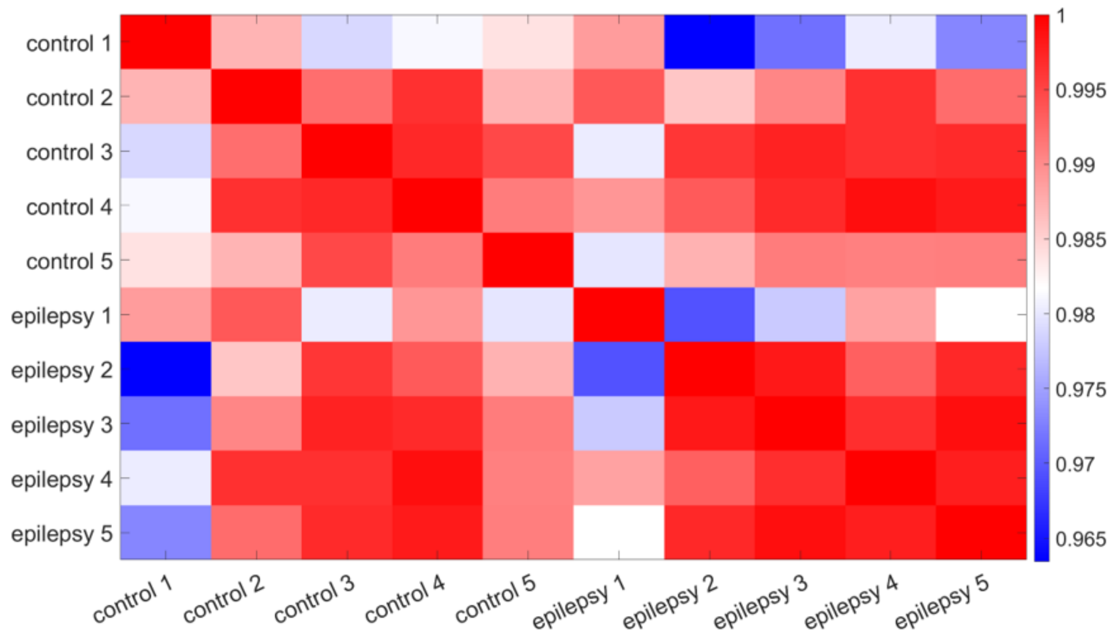

Figure S2. Pearson correlation shows a very high similarity (>0.95) between all samples.

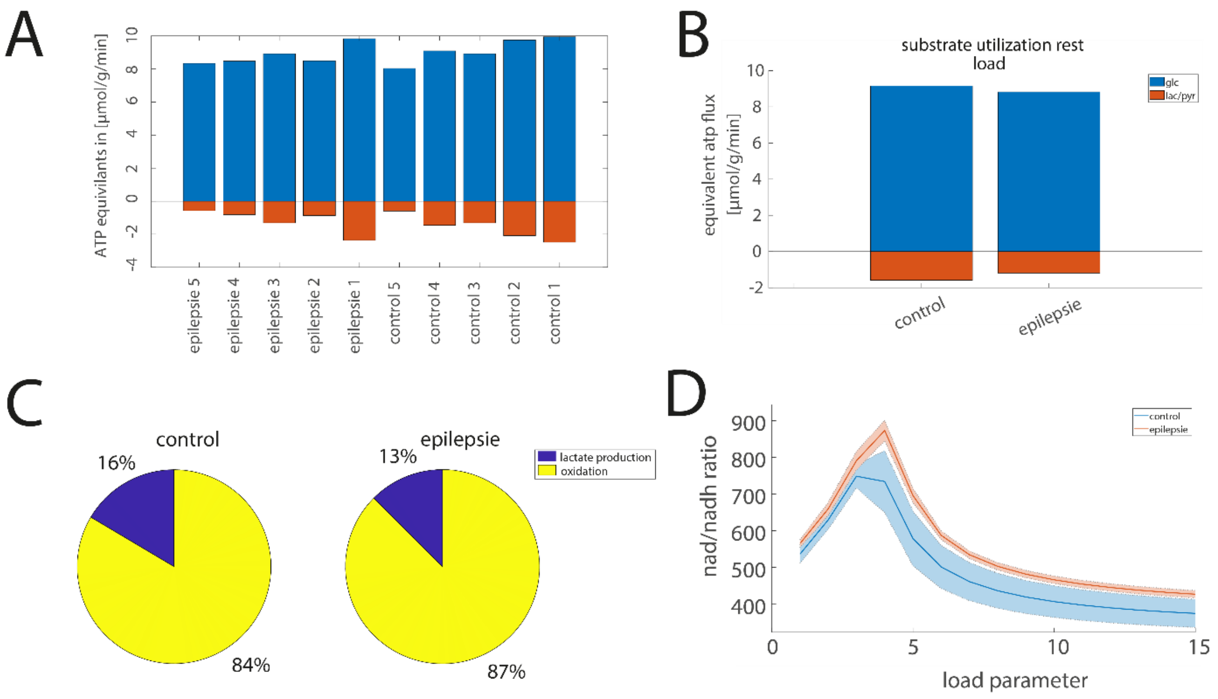

Figure S3. Metabolic differences between control slices (control) and slices subjected to epileptiform activity for 8 h (epilepsy). (**A+B**) Glucose utilization and lactate production under resting conditions for each slice (A) and mean of the two groups (B). (**C**) The relative contribution of glucose utilization of oxidation and lactate production under resting conditions. All numbers are given in ATP equivalents. (**D**) Cytosolic NAD/NADH ratio from rest to maximal ATP demand for control (blue) and epileptic slices (red). Solid lines depict the means and shaded area standard deviation of all slices.

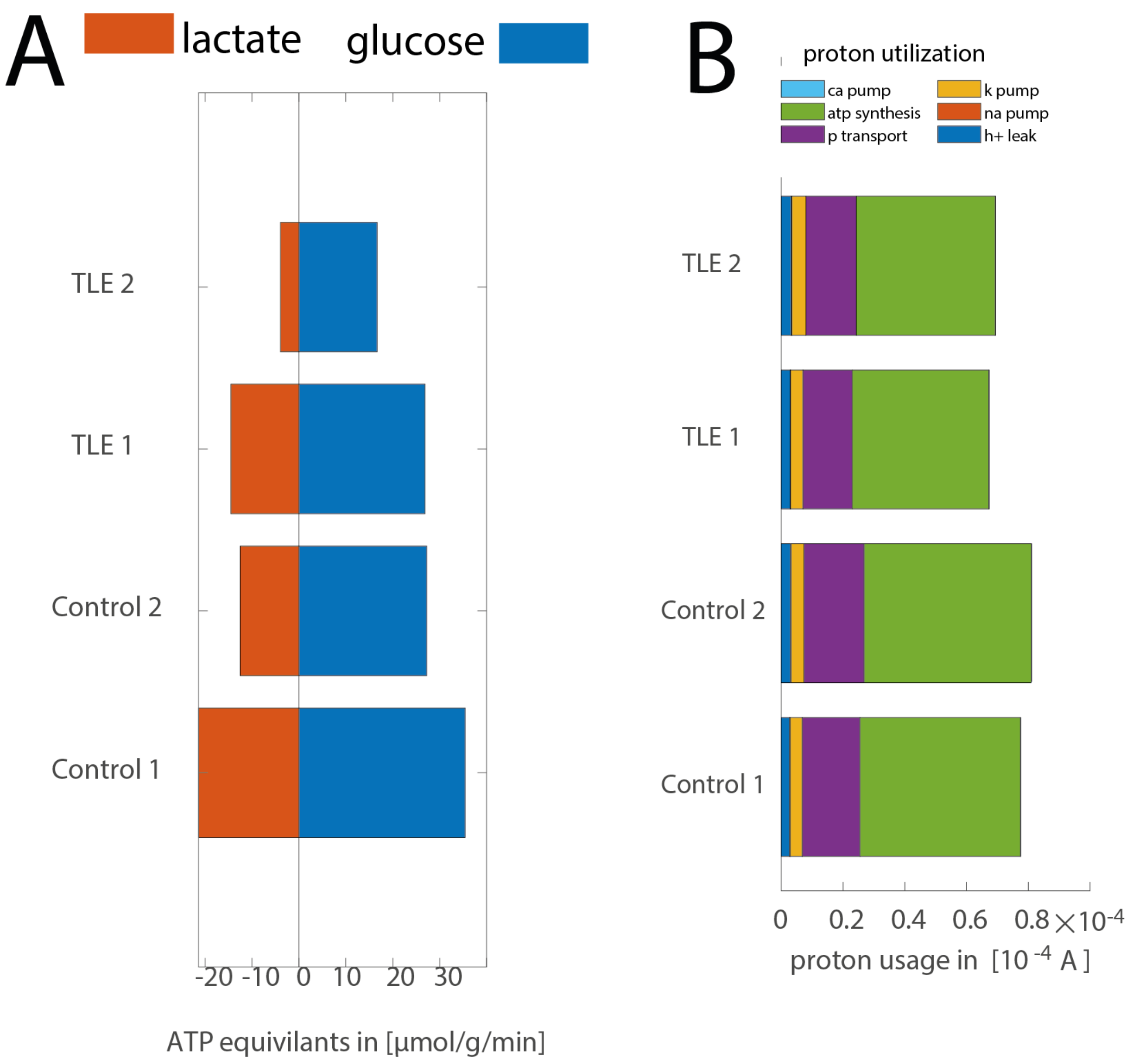

Figure S4. Metabolic alterations in patients with temporal lobe epilepsy (TLE). (**A**) Glucose utilization (red) and lactate production rates (blue) at maximal ATP production are given in ATP equivalents for each sample. As part of the glucose is converted to lactate, lactate is released (negative flux), while glucose is taken up (positive flux). (**B**) Proton utilization for the different mitochondrial membrane processes for each sample given as currents. The size of the different colored bars indicates the magnitude of the different processes (light blue: calcium pumping; green: FOF1-ATPase; purple: phosphate transport; yellow: potassium pumping; red: sodium pumping; dark blue: proton leakage).

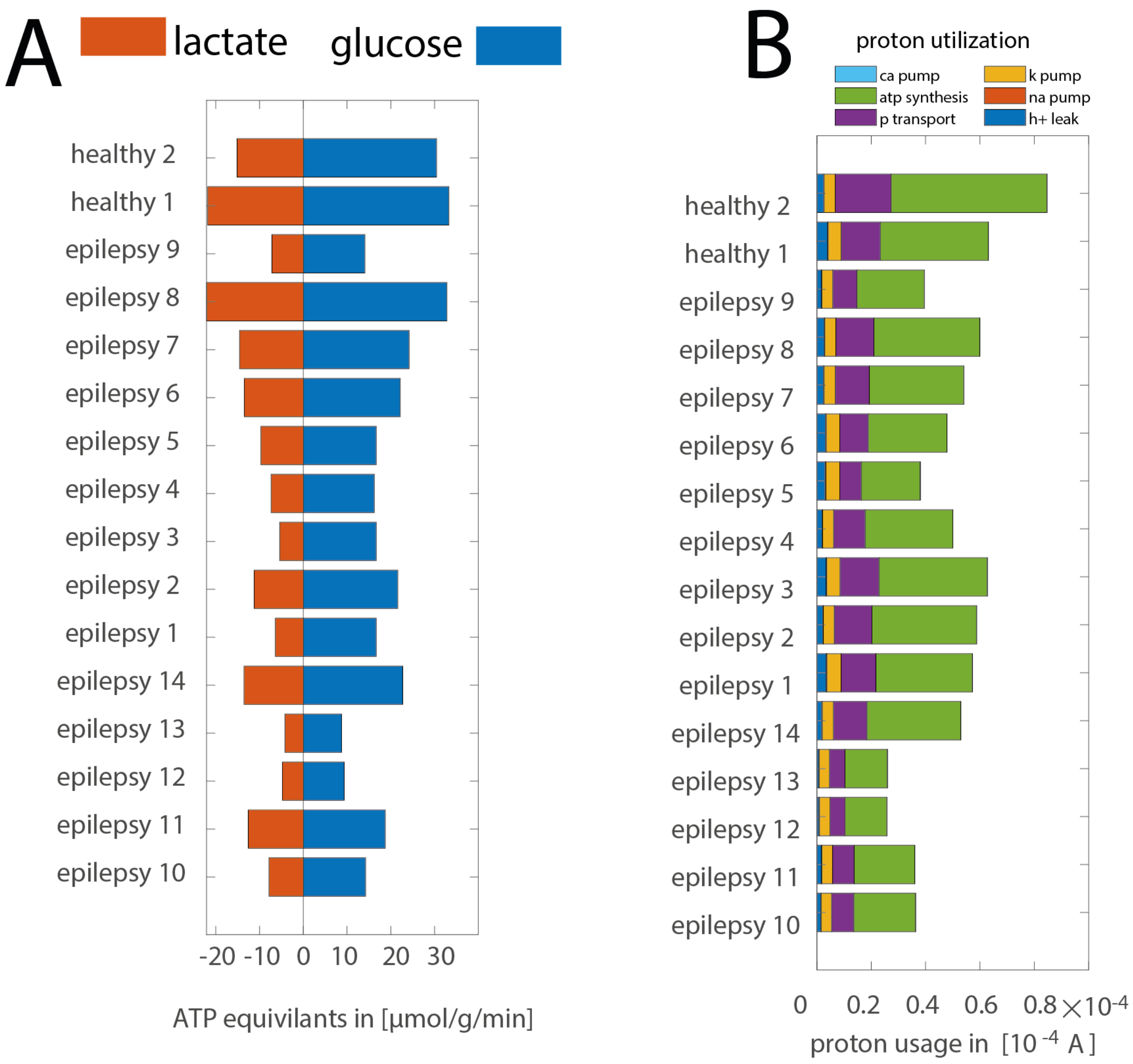

Figure S5. Metabolic alterations in human epileptic tissue. (**A**) Glucose utilization (red) and lactate production rates (blue) at maximal ATP production are given in ATP equivalents for each sample. As part of the glucose is converted to lactate, lactate is released (negative flux), while glucose is taken up (positive flux). (**B**) Proton utilization for the different mitochondrial membrane processes for each sample given as currents. The size of the different colored bars indicates the magnitude of the different processes (light blue: calcium pumping; green: FOF1-ATPase; purple: phosphate transport; yellow: potassium pumping; red: sodium pumping; dark blue: proton leakage).
